# Relationship Between Vitamin D Status, Pain Severity, and Functional Disability in Chronic Low Back Pain

**DOI:** 10.7759/cureus.105376

**Published:** 2026-03-17

**Authors:** SK Imran Ali, Soumya Ghosh, Tuhin Purkayastha, Siddharth Keswani

**Affiliations:** 1 Orthopedics, ICARE Institute of Medical Sciences and Research and Dr. Bidhan Chandra Roy Hospital, Haldia, Haldia, IND; 2 Orthopedics and Trauma, ICARE Institute of Medical Sciences and Research and Dr. Bidhan Chandra Roy Hospital, Haldia, Haldia, IND

**Keywords:** chronic low back pain (clbp), functional outcome, hypovitaminosis d, pain assessment, tertiary care center

## Abstract

Background

Chronic low back pain is a leading cause of disability worldwide and is frequently categorized as a chronic non-specific musculoskeletal condition. Vitamin D deficiency is highly prevalent in the Indian population and has been postulated to contribute to musculoskeletal pain and functional impairment, although existing evidence remains inconsistent and inconclusive.

Objectives

The primary objective of this study was to evaluate the relationship between serum vitamin D levels and chronic low back pain. Secondary objectives included assessment of the association between serum vitamin D levels, pain severity, and functional disability among affected patients.

Methods

This was a hospital-based observational cross-sectional study conducted at a single tertiary care center. Adult patients presenting with chronic low back pain of more than 12 weeks’ duration were enrolled after meeting predefined inclusion and exclusion criteria. Serum 25-hydroxyvitamin D levels were measured and categorized according to standard reference ranges. Pain severity was assessed using the Numeric Pain Rating Scale, and functional disability was evaluated using the modified Oswestry Disability Questionnaire. Statistical analysis was performed to determine associations between vitamin D status, pain intensity, and functional disability, with significance set at an appropriate confidence level.

Results

A high prevalence of vitamin D deficiency and insufficiency was observed among patients with chronic low back pain. Lower serum vitamin D levels were significantly associated with increased pain severity and higher disability scores. Patients with deficient vitamin D levels demonstrated greater functional impairment compared to those with sufficient levels. No statistically significant correlations were identified between serum vitamin D concentration, pain intensity, and functional disability scores.

Conclusions

Vitamin D deficiency is common among patients with chronic low back pain and is significantly associated with greater pain severity and functional disability. These findings suggest that assessment of vitamin D status may be clinically relevant in the evaluation of patients presenting with chronic low back pain, particularly in regions with a high prevalence of hypovitaminosis D.

## Introduction

Chronic low back pain represents a significant global health challenge and is recognized as one of the leading causes of disability, work absenteeism, and increased health care utilization worldwide [1]. Epidemiological data suggest that nearly one in 10 individuals develops chronic low back pain annually, and the condition remains among the most frequent reasons for consultation in primary care settings [2]. Clinically, chronic low back pain is most often categorized as chronic non-specific musculoskeletal pain, defined by pain persisting for longer than 12 weeks without an identifiable structural, inflammatory, infective, or neoplastic cause [3]. The heterogeneous nature of this condition, influenced by a complex interplay of biological, psychological, and social factors, contributes to challenges in diagnosis, management, and prediction of treatment outcomes [4].

In recent years, attention has increasingly shifted toward potentially modifiable biological factors that may contribute to chronic musculoskeletal pain syndromes [5]. Among these, vitamin D has gained particular interest due to its well-established role in calcium and phosphate metabolism, bone health, and skeletal muscle function [6]. Emerging evidence indicates that vitamin D may also influence neuromuscular coordination, inflammatory mediators, immune responses, and pain signaling pathways, thereby suggesting a possible association with chronic pain mechanisms beyond its classical effects on bone metabolism [7,8].

Vitamin D deficiency is widely prevalent across the globe and is now regarded as a major public health concern. Paradoxically, high rates of hypovitaminosis D have been reported even in regions with abundant sunlight, including developing countries [9]. In the Indian subcontinent, studies have reported prevalence rates of vitamin D deficiency ranging from 50% to as high as 95%, a phenomenon attributed to factors such as reduced sun exposure, darker skin pigmentation, inadequate dietary intake, cultural practices, and increasingly sedentary lifestyles [10,11]. The coexistence of a high burden of chronic low back pain and widespread vitamin D deficiency has therefore prompted considerable interest in exploring a potential association between these two conditions.

A number of observational and interventional studies have examined the relationship between serum vitamin D levels and musculoskeletal pain, including chronic low back pain, although the findings remain inconsistent [12-14]. While some studies have reported associations between lower serum 25-hydroxyvitamin D levels, and greater pain intensity, impaired functional capacity, and reduced quality of life, others have failed to demonstrate a meaningful correlation [15-17]. Similarly, interventional trials assessing the effects of vitamin D supplementation in patients with chronic low back pain have produced variable results. Differences in study design, supplementation protocols, outcome measures, baseline vitamin D status, and population characteristics have limited the comparability and generalizability of these findings [18-20].

These inconsistencies highlight important gaps in the current understanding of the role of vitamin D in chronic low back pain, particularly in populations with a high baseline prevalence of hypovitaminosis D [21]. Moreover, data derived from single-center studies conducted in tertiary care settings in India remain relatively sparse. The relationship between serum vitamin D levels, pain severity, and functional disability in this specific clinical context has not been sufficiently explored [22].

Despite the growing interest in vitamin D as a potential contributor to musculoskeletal pain, an important distinction must be made between the prevalence of vitamin D deficiency in patients with chronic low back pain and the existence of a clinically meaningful association with pain severity or disability. In populations where hypovitaminosis D is highly prevalent, the coexistence of vitamin D deficiency and chronic pain conditions may occur frequently without necessarily indicating a causal relationship [6,12]. Therefore, careful evaluation of both the prevalence of deficiency and its potential association with clinical outcomes is necessary to better understand its relevance in chronic low back pain. Clarifying this distinction may help determine whether vitamin D deficiency represents a modifiable risk factor influencing symptom severity or simply a commonly coexisting condition within affected populations [12,15].

Against this background, the present study was designed to evaluate the prevalence between serum vitamin D levels and chronic low back pain among patients attending a tertiary care center. Additionally, the study sought to examine the association between vitamin D status, pain intensity, and functional disability, with the aim of contributing region-specific evidence to an area characterized by ongoing clinical uncertainty [23].

## Materials and methods

This hospital-based observational cross-sectional study was conducted in the Department of Orthopedics at a tertiary care teaching hospital in eastern India over a defined study period. The study was designed to evaluate the association between serum vitamin D levels and chronic low back pain, as well as the relationship between vitamin D status, pain severity, and functional disability [1].

Adult patients aged 18 years and above presenting to the outpatient department with chronic low back pain of more than 12 weeks’ duration were considered eligible for inclusion. Chronic low back pain was defined as pain localized to the lumbosacral region, with or without radiation, persisting for at least three months [2]. Patients were excluded if they had identifiable specific causes of low back pain, such as trauma, infection, malignancy, inflammatory spondyloarthropathy, vertebral fractures, metabolic bone disease other than vitamin D deficiency, neurological disorders, pregnancy, prior spinal surgery, or current use of vitamin D supplementation or medications affecting vitamin D metabolism [3,4].

After obtaining informed written consent, demographic and clinical data were collected, including age, sex, occupation, duration of symptoms, and relevant medical history. Pain severity was assessed using the Numeric Pain Rating Scale, a validated tool in which patients rate their pain intensity on a scale from 0 to 10, with higher scores indicating greater pain intensity [20]. Functional disability was evaluated using the modified Oswestry Disability Questionnaire, which measures the impact of low back pain on daily activities and expresses disability as a percentage score, with higher scores indicating greater functional impairment [21]. The English version of the Oswestry Disability Index (ODI) was used in this study under the licensed module ODI AU2.1b, which corresponds to the English (UK) version of the questionnaire. Permission for the use of this version was obtained through the official ODI licensing system. As the study participants were able to understand English, the questionnaire was administered in its licensed English format. To support the appropriateness of using the ODI in the Indian population, the methodology also cites the study by Nishant et al., which reported a modified English and Hindi version of the Oswestry Disability Index validated among Indian patients with low back pain. This reference is included only to demonstrate the applicability and prior validation of the ODI in the Indian clinical context [22].

Venous blood samples were collected from all participants under aseptic precautions for estimation of serum 25-hydroxyvitamin D levels, which is the accepted biochemical marker for assessing vitamin D status [7]. Serum vitamin D levels were categorized according to standard reference ranges into deficiency, insufficiency, sufficiency, and toxicity, based on established clinical guidelines [8]. All laboratory analyses were performed in the institutional laboratory using standardized assay techniques.

Data were entered into a structured database and analyzed using standard statistical software. Descriptive statistics were used to summarize demographic variables, vitamin D status, pain scores, and disability scores. Continuous variables were expressed as means with standard deviations, while categorical variables were expressed as frequencies and percentages. Appropriate statistical tests were applied to evaluate associations between serum vitamin D levels, pain severity, and functional disability. Correlation and comparative analyses were performed as indicated, with a predefined level of statistical significance [9,10].

Ethical approval for the study was obtained from the Institutional Ethics Committee, ICARE Institute of Medical Sciences and Research (IEC-IIMSAR), Haldia, West Bengal, prior to commencement of data collection (Reference No.: IIMSAR-Haldia/IEC/June 2023/15). The study protocol was reviewed and approved in the committee meeting held on June 5, 2023. The study was conducted in accordance with the principles of the Declaration of Helsinki and applicable institutional and national ethical guidelines. Written informed consent was obtained from all participants before enrollment, and confidentiality of patient information was strictly maintained throughout the study.

## Results

A total of 75 patients diagnosed with chronic low back pain were included in the final analysis after applying predefined inclusion and exclusion criteria. All participants had symptoms persisting for more than 12 weeks at the time of presentation. The study population consisted of adult patients aged 18 years and above who attended the orthopedics outpatient department of a tertiary care teaching hospital during the study period [1].

The age distribution of the study population demonstrated that the majority of patients belonged to the 35-50 years age group, accounting for 39 (52.0%) patients. Patients aged between 18 and 34 years constituted 18 (24.0%) cases, while patients older than 50 years accounted for the remaining 18 (24.0%) cases. The distribution of age groups did not demonstrate a statistically significant association with vitamin D status at presentation. Mean age was higher in the vitamin D deficiency group compared to the insufficiency group; however, this difference did not reach statistical significance [2]. Detailed demographic characteristics are presented in Table 1.

**Table 1:** Demographic characteristics of the study population This table summarizes the demographic profile of the study participants, including age distribution and sex composition. Values are presented as number and percentage. The male-to-female ratio is also shown.

Variable	Category	Number (n)	Percentage (%)
Age (years)	18–34	18	24
	35–50	39	52
	>50	18	24
Sex	Male	21	28
	Female	54	72
Male-to-female ratio	-	0.4:1	-

Sex distribution analysis revealed a predominance of female patients in the study cohort. Of the total 75 patients, 54 (72.0%) were female and 21 (28.0%) were male, resulting in a male-to-female ratio of 0.4:1. There was no statistically significant association between sex and vitamin D status at presentation. Both male and female patients demonstrated a high prevalence of hypovitaminosis D [3].

Serum 25-hydroxyvitamin D estimation revealed a markedly high prevalence of low vitamin D levels among patients with chronic low back pain. Vitamin D deficiency, defined as serum levels less than 20 ng/mL, was identified in 52 (69.3%) patients. Vitamin D insufficiency, defined as serum levels between 20 and 30 ng/mL, was observed in 23 (30.7%) patients. No patient in the study population had sufficient serum vitamin D levels, defined as levels greater than 30 ng/mL. The overall mean serum vitamin D level at presentation for the entire cohort was 16.69 ± 5.85 ng/mL. The distribution of vitamin D status is summarized in Table 2 [4].

**Table 2:** Distribution of serum vitamin D status at presentation This table shows the distribution of patients according to serum 25-hydroxyvitamin D levels at the time of presentation. Vitamin D status is categorized as deficiency, insufficiency, or sufficiency based on standard reference ranges. Values are expressed as number and percentage.

Vitamin D Status	Serum Level (ng/mL)	Number (n)	Percentage (%)
Deficiency	<20	52	69.3
Insufficiency	20–30	23	30.7
Sufficiency	>30	0	0

Pain severity was assessed using the Numeric Pain Rating Scale at the time of presentation. The overall pain scores ranged from 3 to 8 across the study population. In the vitamin D deficiency group, the mean pain score was 5.13 with a standard deviation of 1.29. In the vitamin D insufficiency group, the mean pain score was 5.30 with a standard deviation of 1.21. The difference in mean pain scores between the deficiency and insufficiency groups was not statistically significant, with a p-value of 0.4243. Pain severity distribution across vitamin D categories is detailed in Table 3 [5].

**Table 3:** Comparison of pain severity scores by vitamin D status using the independent t-test Pain severity was assessed using the NPRS [20]. Values are presented as mean ± standard deviation. Comparison between groups was performed using an independent t-test. NPRS, Numeric Pain Rating Scale

Vitamin D Status	n	Mean NPRS Score	Standard Deviation	t-Value	p-Value
Deficiency	52	5.13	1.29		
Insufficiency	23	5.3	1.21	−0.80	0.4243

When pain scores were categorized based on severity, moderate pain was the most commonly reported category among patients in both vitamin D deficiency and insufficiency groups. A higher proportion of patients in the deficiency group reported moderate pain compared to the insufficiency group; however, this difference did not demonstrate statistical significance. Severe pain was less commonly reported and was observed in both groups without a significant difference in distribution [6].

Functional disability was evaluated using the modified Oswestry Disability Questionnaire. Disability scores ranged from 15 to 34 across the study population. In the vitamin D deficiency group, the mean disability score was 21.62 with a standard deviation of 5.10. The median disability score in this group was 21. In the vitamin D insufficiency group, the mean disability score was 23.43 with a standard deviation of 5.03, with a median score of 22. The difference in disability scores between the two groups was not statistically significant, with a p-value of 0.1565. Functional disability data are presented in Table 4 [7].

**Table 4:** Comparison of functional disability scores by vitamin D status using the independent t-test Functional disability was assessed using the MODQ [21]. Values are expressed as mean ± standard deviation. Independent t-test was used for comparison between groups. MODQ, modified Oswestry Disability Questionnaire

Vitamin D Status	n	Mean MODQ Score	Standard Deviation	t-Value	p-Value
Deficiency	52	21.62	5.1	-	-
Insufficiency	23	23.43	5.03	−1.43	0.1565

Based on the modified Oswestry Disability Questionnaire categorization, moderate disability was the most common level observed in both vitamin D deficiency and insufficiency groups. Mild disability was observed in a smaller proportion of patients, while severe disability was less frequent. No patient in the study population was classified as completely disabled at the time of presentation [8].

Socioeconomic status analysis demonstrated a statistically significant association with vitamin D status. Among patients with vitamin D deficiency, six (11.5%) patients belonged to the lower socioeconomic class, 32 (61.5%) patients belonged to the lower middle class, and 14 (26.9%) patients belonged to the upper middle class. In contrast, among patients with vitamin D insufficiency, two (8.7%) patients were from the lower class, seven (30.4%) patients were from the lower middle class, and 14 (60.9%) patients were from the upper middle class. The association between socioeconomic class and vitamin D status was statistically significant, with a p-value of 0.0182. These findings are summarized in Table 5 [9].

**Table 5:** Association between socioeconomic status and vitamin D status using the chi-square test The association between socioeconomic status and vitamin D status was analyzed using the chi-square test. Values are expressed as number and percentage. socioeconomic status and vitamin D status. Chi-square value (χ²) = 8.05; p-value = 0.0182

Socioeconomic Class	Vitamin D Deficiency, n (%)	Vitamin D Insufficiency, n (%)
Lower	6 (11.5)	2 (8.7)
Lower middle	32 (61.5)	7 (30.4)
Upper middle	14 (26.9)	14 (60.9)

Body mass index was higher in the vitamin D deficiency group compared to the insufficiency group, and this difference was statistically significant. However, body mass index values did not demonstrate a statistically significant association with pain severity or disability scores within the scope of this analysis [10].

Duration of pain, measured in months, was longer in the vitamin D insufficiency group compared to the deficiency group. This difference did not reach statistical significance. The duration of symptoms ranged from 3 months to more than 12 months across the study population [11].

Correlation analysis was performed to assess the relationship between serum vitamin D levels and pain severity. The Pearson correlation coefficient between serum vitamin D levels and Numeric Pain Rating Scale scores was 0.092. This correlation was weak and did not reach statistical significance. Similarly, correlation analysis between serum vitamin D levels and modified Oswestry Disability Questionnaire scores yielded a Pearson correlation coefficient of 0.089, indicating a weak positive correlation that was not statistically significant [12].

No statistically significant association was observed between vitamin D deficiency status and severity of chronic low back pain based on pain score categorization. Additionally, no statistically significant association was identified between vitamin D deficiency status and functional disability levels among the study participants. These findings were consistent across subgroup analyses stratified by age and sex [13].

All statistical analyses were conducted using standard statistical methods, with p-values less than or equal to 0.05 considered statistically significant. Results were summarized using means, standard deviations, medians, frequencies, and percentages, as appropriate [14].

## Discussion

This hospital-based cross-sectional study examined the relationship between serum vitamin D levels and chronic low back pain in adult patients attending a tertiary care center. The primary aim was to determine whether hypovitaminosis D is associated with chronic low back pain, while secondary objectives focused on evaluating its relationship with pain severity and functional disability. The results of the present study indicate a high prevalence of vitamin D deficiency and insufficiency among patients with chronic low back pain. However, no statistically significant association was observed between serum vitamin D levels and either pain intensity or functional disability, with the exception of a significant relationship identified between vitamin D status and socioeconomic class [1].

Chronic low back pain continues to be one of the leading contributors to disability worldwide and poses a substantial burden on healthcare systems, particularly in low- and middle-income countries [2]. The condition is inherently multifactorial, arising from a complex interaction of biomechanical, psychological, social, and biological factors, which complicates both its evaluation and management [3]. Within this multifaceted framework, vitamin D deficiency has been proposed as a potentially modifiable biological contributor to musculoskeletal pain, owing to its established roles in bone metabolism, muscle strength, and neuromuscular coordination [4]. Consequently, increasing attention has been directed toward understanding the potential association between vitamin D status and chronic pain conditions, including chronic low back pain [5].

In the present study, vitamin D deficiency was identified in 69.3% of patients, while the remaining 30.7% demonstrated vitamin D insufficiency. Notably, none of the participants had sufficient serum vitamin D levels. These findings are in agreement with existing literature reporting a high prevalence of hypovitaminosis D in the Indian population, even in regions with adequate sunlight exposure [6,7]. Multiple factors, including reduced sun exposure, increased skin pigmentation, dietary inadequacy, urbanization, and lifestyle modifications, have been implicated in the widespread occurrence of vitamin D deficiency in this population [8]. While the high prevalence observed in this study underscores the frequent coexistence of hypovitaminosis D and chronic low back pain, prevalence alone does not imply a causal relationship or clinical significance [9].

Assessment of pain severity using the Numeric Pain Rating Scale revealed no statistically significant difference between patients with vitamin D deficiency and those with vitamin D insufficiency. Mean pain scores were comparable across both groups, and the distribution of pain severity categories did not demonstrate a meaningful association with vitamin D status. These observations are consistent with several previous studies that failed to establish a significant correlation between serum vitamin D levels and pain intensity in patients with chronic low back pain [10,11]. Kumar et al. similarly reported a high prevalence of vitamin D deficiency in patients with chronic low back pain without identifying a significant relationship between vitamin D levels and pain severity, suggesting that while hypovitaminosis D is common, it may not directly influence pain intensity [12].

Functional disability, evaluated using the modified Oswestry Disability Questionnaire, also did not show a statistically significant association with vitamin D status in this study. Although slightly higher mean disability scores were observed in the vitamin D insufficiency group compared to the deficiency group, the difference did not reach statistical significance. These findings suggest that serum vitamin D levels may not independently determine functional impairment in individuals with chronic low back pain. Comparable results have been reported in earlier studies, where functional outcomes were not significantly influenced by vitamin D status [13]. In contrast, some interventional studies have suggested improvements in functional outcomes following vitamin D supplementation, highlighting the considerable heterogeneity in study designs, supplementation regimens, baseline vitamin D levels, and outcome measures across the literature [14].

The absence of a significant association between vitamin D levels and either pain severity or functional disability in the present study may be explained by several factors. Chronic low back pain is a complex condition in which pain perception is shaped by an intricate interaction of biological, psychological, and social influences [15]. Vitamin D deficiency may coexist with chronic pain without serving as a direct causative factor. Additionally, reduced physical activity resulting from chronic pain may limit sun exposure, thereby contributing to lower vitamin D levels and creating an apparent association without a causal relationship [16]. Furthermore, central sensitization, psychosocial stressors, and maladaptive pain coping mechanisms play a substantial role in chronic pain syndromes and may overshadow the potential effects of nutritional deficiencies on pain and disability [17].

The weak and statistically non-significant correlations observed between serum vitamin D levels and both pain severity and functional disability further support the lack of a strong linear relationship between these variables. The minimal Pearson correlation coefficients observed in this study are consistent with previous observational research reporting weak or inconsistent associations between vitamin D status and musculoskeletal pain outcomes [18]. These findings suggest that vitamin D levels alone may not serve as a reliable predictor of pain intensity or functional impairment in patients with chronic low back pain.

A notable finding of this study was the statistically significant association between socioeconomic status and vitamin D deficiency. Patients belonging to lower and lower middle socioeconomic groups were more frequently represented in the vitamin D deficiency category, whereas a higher proportion of patients from the upper middle socioeconomic group were observed in the insufficiency category. This observation aligns with existing evidence indicating that socioeconomic factors influence nutritional status, sun exposure, dietary quality, and access to healthcare services, all of which may affect vitamin D levels [19]. Individuals from lower socioeconomic backgrounds may have limited access to vitamin D-rich foods, reduced opportunities for outdoor activity, and decreased healthcare access, thereby increasing the risk of hypovitaminosis D [20].

The study population demonstrated a predominance of female patients, which is consistent with prior reports indicating a higher prevalence of chronic low back pain among women [21]. Proposed explanations for this sex difference include hormonal influences, variations in pain perception, occupational and domestic roles, and differences in healthcare-seeking behavior [23]. However, in the present study, sex was not significantly associated with vitamin D status, pain severity, or functional disability, suggesting that the observed female predominance did not confound the primary study outcomes [24].

Several limitations of the present study must be acknowledged. The relatively small sample size may have limited the ability to detect subtle associations between serum vitamin D levels and clinical outcomes. The single-center design restricts the generalizability of the findings to other populations and settings. Additionally, the cross-sectional nature of the study precludes conclusions regarding temporal relationships or causality between vitamin D deficiency and chronic low back pain [25]. The absence of a control group without low back pain further limits interpretation, as it remains unclear whether the observed prevalence of hypovitaminosis D is specific to patients with chronic low back pain or reflective of the general population [26].

Despite these limitations, the study offers valuable region-specific insights into vitamin D status among patients with chronic low back pain in a tertiary care setting. The use of validated and standardized tools for assessing pain and functional disability enhances the reliability of the findings. Importantly, the study contributes to the growing body of evidence suggesting that although vitamin D deficiency is highly prevalent among patients with chronic low back pain, its role as an independent determinant of pain severity and functional disability remains uncertain.

From a clinical perspective, these findings suggest that routine screening for vitamin D deficiency in patients with chronic low back pain should be approached with caution. While correction of vitamin D deficiency may offer general musculoskeletal and systemic health benefits, current evidence does not clearly support its use as a targeted intervention for reducing pain intensity or disability in chronic low back pain. Future research involving larger, multicenter, longitudinal studies and well-designed randomized controlled trials is necessary to better define the therapeutic role of vitamin D supplementation in this patient population.

A number of observational studies and systematic analyses have explored the possible link between vitamin D deficiency and chronic low back pain, though the conclusions across the literature remain mixed. In a systematic review and meta-analysis, Zadro et al. evaluated observational research examining serum vitamin D levels in individuals with low back pain and reported considerable variability in findings across studies [27]. While several investigations suggested that lower vitamin D levels may be more common among patients with low back pain, the overall evidence was inconsistent and limited by differences in study populations, diagnostic criteria, and outcome measures. Variability in baseline vitamin D status across geographic regions, as well as methodological differences in pain assessment, further complicates interpretation of these findings. As a result, the current body of evidence does not definitively establish vitamin D deficiency as an independent risk factor for chronic low back pain.

Similar observations have been reported in other clinical investigations. Lodh et al. assessed vitamin D levels in patients presenting with chronic low back pain of unclear etiology and documented a substantial proportion of individuals with hypovitaminosis D within this population [28]. Although these findings suggested that vitamin D deficiency frequently coexists with chronic musculoskeletal pain, the authors noted that the presence of deficiency alone does not necessarily indicate a direct causal relationship. More recently, Alonso-Pérez et al. conducted a systematic review evaluating the association between serum vitamin D levels and chronic musculoskeletal pain in adults and concluded that while vitamin D deficiency is commonly observed among patients with persistent pain syndromes, the strength and consistency of this association vary widely between studies [29]. The results of the present study align with these observations, demonstrating a high prevalence of hypovitaminosis D among patients with chronic low back pain but no statistically significant association between serum vitamin D levels and either pain severity or functional disability. Taken together, these findings further support the concept that chronic low back pain is a multifactorial condition in which nutritional factors such as vitamin D status may coexist with, but do not necessarily determine, clinical severity.

In conclusion, the present study highlights a high prevalence of hypovitaminosis D among patients with chronic low back pain but does not demonstrate a significant association between serum vitamin D levels and pain severity or functional disability. These findings emphasize the multifactorial nature of chronic low back pain and reinforce the need for a comprehensive biopsychosocial approach to its evaluation and management rather than reliance on a single biological marker.

## Conclusions

The present study highlights a high prevalence of hypovitaminosis D among patients presenting with chronic low back pain in a tertiary care setting. Although vitamin D deficiency and insufficiency were commonly observed, no statistically significant association was found between serum vitamin D levels and either pain severity or functional disability. These findings indicate that reduced vitamin D levels may frequently coexist with chronic low back pain without independently influencing clinical severity or functional impairment.

Chronic low back pain is a complex and multifactorial condition, and its assessment and management should therefore adopt a holistic approach that considers biomechanical, psychosocial, and lifestyle-related factors rather than relying on a single biochemical marker. While correction of vitamin D deficiency remains important for overall musculoskeletal and general health, its role as a targeted therapeutic intervention for alleviating pain intensity or improving functional outcomes in chronic low back pain remains uncertain. Further evidence from larger, longitudinal, and interventional studies is required to clarify its potential clinical relevance.
